# Inclisiran-induced LDL-cholesterol reduction with and without concomitant statin therapy: a real-world analysis

**DOI:** 10.1186/s12872-026-06429-w

**Published:** 2026-08-15

**Authors:** Maximilian Seidel, Felix S. Seibert, Moritz Anft, Ulrik Stervbo, Nina Babel, Timm H. Westhoff, Sebastian Bertram

**Affiliations:** Medizinische Klinik I, Universitätsklinik Marien Hospital Herne, Ruhr-Universität Bochum, Hölkeskampring 40, Herne, 44625 Germany

**Keywords:** Inclisiran, LDL-cholesterol, PCSK9-inhibition, Statin therapy

## Abstract

Inclisiran lowers LDL-cholesterol (LDL-C) by inhibiting hepatic PCSK9 synthesis. Whether concomitant statin therapy modifies the magnitude of inclisiran-associated LDL-C reduction in routine clinical practice remains uncertain. In this retrospective single-center observational study, 67 patients treated with inclisiran for at least 9 months were analyzed. Patients previously treated with PCSK9 monoclonal antibodies were excluded. LDL-C values were assessed longitudinally and stratified by concomitant statin therapy. Multivariable linear mixed-effects models were used to evaluate inclisiran-associated percentage LDL-C reduction over time. Thirty-seven patients received concomitant statin therapy and 30 patients did not. Median follow-up was 18 months (IQR 12.5–28). Median LDL-C reduction ranged from approximately − 25% to − 40% across follow-up visits. At 9 months, 10 of 59 patients (16.9%) achieved a ≥ 50% LDL-C reduction. In the primary multivariable longitudinal mixed-effects model, concomitant statin use was not significantly associated with percentage LDL-C reduction (β for no statin vs. statin: 33.1% points; 95% CI − 4.6 to 70.7; *p* = 0.085). Statin intensity was also not significantly associated with LDL-C reduction (β − 0.11 per percentage point of maximum approved dose; 95% CI − 0.60 to 0.37; *p* = 0.644). In an exploratory responder analysis, concomitant statin therapy was associated with higher odds of achieving ≥ 50% LDL-C reduction at 9 months (OR 7.69; 95% CI 1.28–45.45; *p* = 0.026). During follow-up, 18 patients (26.9%) discontinued inclisiran, predominantly due to insufficient LDL-C reduction. In routine clinical practice, neither concomitant statin use nor statin intensity was significantly associated with the magnitude of LDL-C reduction observed during inclisiran therapy. However, responder and attrition sensitivity analyses suggested a possible association between concomitant statin therapy and a more pronounced inclisiran-associated LDL-C response. These findings support the clinical use of inclisiran in heterogeneous real-world populations, while highlighting the potential benefit of maintaining concomitant statin therapy whenever tolerated.

## Introduction

Proprotein convertase subtilisin/kexin type 9 (PCSK9) inhibitors represent an established strategy for lowering low-density lipoprotein cholesterol (LDL-C) in patients at high cardiovascular risk who do not achieve guideline-recommended targets with conventional therapy. Two monoclonal antibodies (evolocumab and alirocumab) and the small-interfering RNA agent inclisiran are currently approved for clinical use. In randomized trials, monoclonal antibodies reduced LDL-C by approximately 60% [1, 2] while inclisiran achieved reductions of around 50% in the ORION program, with the advantage of twice-yearly maintenance dosing [3]. Heterogeneous findings have been reported regarding the influence of concomitant statin therapy on LDL-C reduction with inclisiran. While pooled post-hoc analyses of the ORION-10 and ORION-11 trials did not demonstrate significant differences according to background statin use [4], real-world meta-analyses and the GIN trial suggested greater short-term LDL-C reductions in statin-treated patients [5, 6]. In parallel, accumulating real-world data have described more modest overall LDL-C reductions with inclisiran (approximately 26%–45%) compared with randomized trials, raising questions about treatment response outside controlled settings [5, 6].

Taken together, these heterogeneous and partly conflicting data highlight an important knowledge gap regarding the determinants of inclisiran responsiveness in routine clinical practice. This issue is particularly relevant given that patients eligible for PCSK9-targeted therapies typically represent a population at very high cardiovascular risk, in whom maximal LDL-C lowering is of critical prognostic importance. The apparent discrepancy between randomized trial results and real-world outcomes underscores the need for further investigations focusing on treatment context, background lipid-lowering therapy, and patient-specific factors.

The present study aimed to evaluate inclisiran-associated LDL-C reduction in routine clinical practice according to concomitant statin background. Specifically, we compared longitudinal LDL-C changes in patients treated with inclisiran with versus without concomitant statin therapy and assessed whether statin use or statin intensity was associated with the magnitude of the inclisiran-associated LDL-C response. This study seeks to contribute to a better understanding of how inclisiran performs in everyday practice and how its lipid-lowering potential may be optimized in high-risk populations.

## Methods

We conducted a retrospective observational study to investigate inclisiran-associated LDL-C reduction with versus without concomitant statin therapy in a real-world clinical setting. Patients were eligible for inclusion if they had received inclisiran therapy for at least nine months. Exclusion criteria comprised prior treatment with the PCSK9 monoclonal antibodies alirocumab or evolocumab, as well as age below 18 years. A total of 67 patients who initiated inclisiran therapy between February 2021 and January 2025 were identified. All patients were managed in an ambulatory setting at the Lipidology Center of Competence of the University Hospital Marien Hospital Herne. The clinical visit at which inclisiran was indicated and initiated was defined as baseline. According to the approved dosing regimen, the second dose of inclisiran was administered three months after the initial injection, followed by maintenance dosing every six months thereafter. Consequently, scheduled follow-up visits occurred at approximately 3, 9, 15, 21, 27 and 33 months after baseline. At each visit, lipid parameters and clinical data were collected as part of routine care and subsequently analyzed for this study. LDL-C was measured using a direct enzymatic assay by the central laboratory of the University Hospital Marien Hospital Herne. Blood sampling was performed in the morning in a specialized lipid clinic. All patients were fasting at the time of blood collection and sampling conditions were highly standardized across visits.

In Germany, prescription of inclisiran within the public health insurance system is restricted by national reimbursement regulations. In clinical practice, inclisiran was prescribed for patients with established stenosing atherosclerotic cardiovascular disease in secondary prevention who failed to achieve LDL-C targets despite maximally tolerated therapy with statins, ezetimibe, and bempedoic acid, as well as patients with familial hypercholesterolemia.

Data distribution was assessed for normality. Continuous variables are presented as median with interquartile range or mean ± standard deviation, as appropriate. Categorical variables are reported as numbers and percentages. Group comparisons were performed using Mann–Whitney U tests for continuous variables and Fisher’s exact tests for categorical variables.

Inclisiran-associated percentage LDL-C reduction over time was analyzed using a multivariable linear mixed-effects model. Fixed effects included follow-up time, concomitant statin use at each visit, statin intensity, baseline LDL-C, age, sex, familial hypercholesterolemia, concomitant ezetimibe use, concomitant bempedoic acid use, and changes in background lipid-lowering therapy compared with the previous visit. Medication variables were coded at each visit to account for treatment changes over time.

Statin intensity was calculated as the prescribed daily dose expressed as a percentage of the maximum approved daily dose according to the prescribing information. In addition, statin stability during follow-up was assessed by recording statin switches and dose reductions.

Responder analysis was performed separately using achievement of ≥ 50% LDL-C reduction at 9 months as a clinically relevant binary endpoint. Responder rates were compared between patients with and without concomitant statin therapy using Fisher’s exact test. Because only 10 patients achieved the responder endpoint, the primary multivariable logistic regression model for the 9-month responder endpoint was deliberately restricted to three clinically relevant covariates to reduce the risk of overfitting: concomitant statin use, baseline LDL-C, and familial hypercholesterolemia. An extended exploratory logistic regression model additionally including age, sex, ezetimibe use, and bempedoic acid use was performed as a sensitivity analysis.

To assess the potential impact of attrition bias due to discontinuation of inclisiran, a sensitivity analysis was performed after excluding patients who discontinued inclisiran during follow-up. The same multivariable linear mixed-effects model was then repeated in this restricted cohort.

A two-sided *p* value < 0.05 was considered statistically significant. The study was conducted in accordance with the Declaration of Helsinki and was approved by the local ethics committee/institutional review board of the Ruhr-University Bochum (approval number: 15-5279). Written informed consent was obtained from all participants prior to inclusion in the study.

## Results

Baseline characteristics stratified by concomitant statin therapy are shown in Table 1. Patients receiving concomitant statin therapy were younger than patients without statin therapy (61.0 [57.0–67.0] vs. 65.5 [60.75–70.25] years; *p* = 0.025). HbA1c was also higher in the statin group (5.90 [5.60–6.10] % vs. 5.60 [5.40–5.83] %; *p* = 0.013). Sex distribution, body mass index, follow-up duration, baseline LDL-C, renal function, concomitant ezetimibe use, bempedoic acid use and comorbidity profiles were comparable between groups.

**Table 1:** Epidemiological and clinical characteristics of the study population stratified by concomitant statin therapy

	With Statin Therapy (*n* = 37)	Without Statin Therapy (*n* = 30)	*p*
Female	20 (54.1%)	17 (56.7%)	> 0.999
Age [years]	61.0 (57.0–67.0)	65.5 (60.75–70.25)	0.025
BMI [kg/m²]	26.5 (23.25–29.75)	26.0 (24.0-29.5)	0.870
Follow-up [Months]	15 (12.5–27.5)	21 (12-31.3)	0.738
Baseline LDL-C [mg/dL]	107.5 (91.75–133.8)	128.5 (94.75–186.8)	0.160
Creatinine [mg/dL]	0.95 (0.80–1.10)	1.00 (0.75–1.20)	0.613
eGFR [mL/min/1.73 m²]	76.5 (66.0-89.75)	71.0 (58.5–89.5)	0.248
HbA1c [%]	5.90 (5.60–6.10)	5.60 (5.40-5.825)	0.013
Number of antihypertensive drugs	2 (1–2)	1.5 (1–2)	0.883
Number of anti-diabetic drugs	0 (0–0)	0 (0–0)	0.657
Comorbidities			
Chronic Kidney Disease	8 (21.6%)	4 (13.3%)	0.525
Coronary Artery Disease	28 (75.7%)	23 (76.7%)	> 0.999
Hypertension	25 (67.6%)	19 (63.3%)	0.798
Diabetes	4 (10.8%)	8 (26.7%)	0.117
Stroke	2 (5.4%)	2 (6.7%)	> 0.999
Peripheral Artery Disease	8 (21.6%)	1 (3.3%)	0.120
Malignancy	1 (2.7%)	2 (6.7%)	0.583
Smoking	8 (21.6%)	4 (13.3%)	0.525
Familial Hypercholesterolemia	9 (24.3%)	8 (26.7%)	> 0.999
Rosuvastatin	17 (45.9%)	/	/
Atorvastatin	17 (45.9%)	/	/
Simvastatin	1 (2.7%)	/	/
Pravastatin	1 (2.7%)	/	/
Fluvastatin	1 (2.7%)	/	/
Ezetimibe	28 (75.7%)	17 (56.7%)	0.122
Bempedoic acid	21 (56.8%)	18 (60.0%)	0.809
Primary prevention	5 (13.5%)	5 (16.7%)	0.743
Secondary prevention	32 (86.5%)	25 (83.3%)	

Continuous variables are presented as median with interquartile range and were compared using the Mann–Whitney U test. Categorical variables are presented as *n* (%) and were compared using Fisher’s exact test. A two-sided *p* value < 0.05 was considered statistically significant

*BMI* Body mass index, *CKD* Chronic kidney disease, *eGFR* estimated glomerular filtration rate, *HbA1c* glycated hemoglobin, *LDL-C* Low-density lipoprotein cholesterol

At 9 months, 10 patients (14.9%) achieved their respective LDL-C targets. During follow-up, 18 patients (26.9%) discontinued inclisiran, predominantly due to insufficient LDL-C reduction. Among 11 patients with available follow-up after switching to PCSK9 monoclonal antibodies, 6 (54.5%) achieved LDL-C target levels within 3 months.

In the overall cohort, median LDL-C reduction ranged between − 25% and − 40% across follow-up visits. During follow-up, 35 patients (52.2%) experienced changes in background lipid-lowering therapy. Background lipid-lowering therapy and changes compared with the previous visit were incorporated into the longitudinal analyses. Changes in background lipid-lowering therapy were not significantly associated with percentage LDL-C reduction in the multivariable mixed-effects model.

**Fig. 1:**
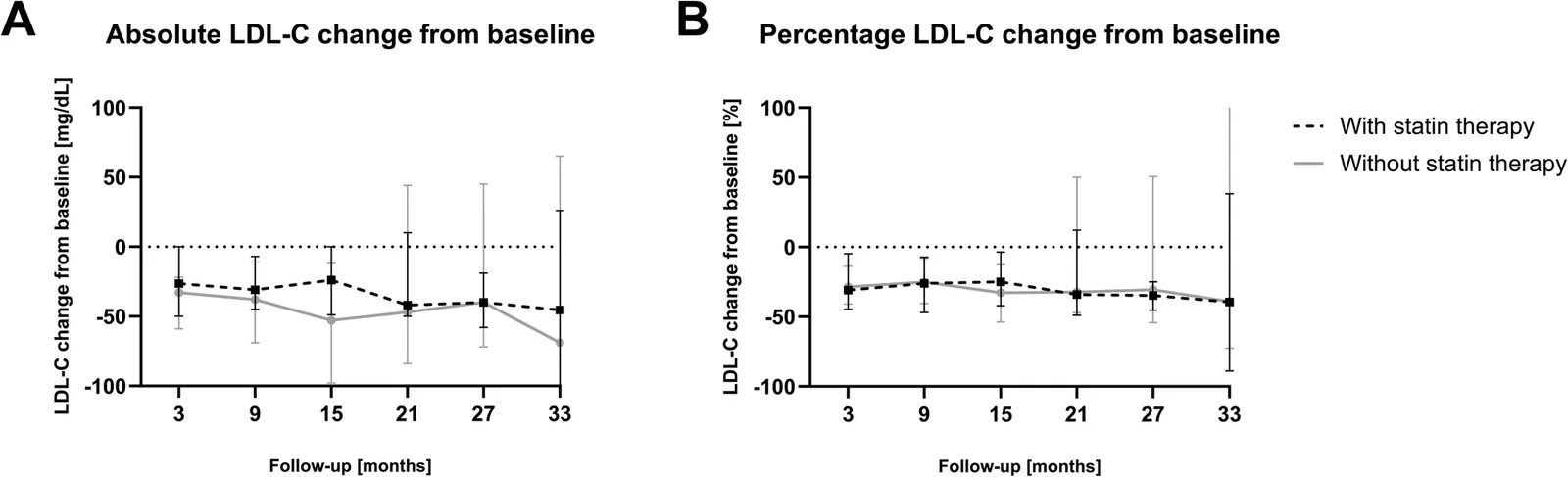
Longitudinal LDL-C changes stratified by concomitant statin therapy. **A ** Absolute LDL-C change from baseline. **B** Percentage LDL-C change from baseline. Data are shown as medians with 95% confidence intervals. Between-group comparisons at each follow-up visit were performed using the Mann–Whitney U test. Negative values indicate LDL-C reduction. No statistically significant between-group differences were observed for either absolute or percentage LDL-C change from baseline at any follow-up time point. Numbers of patients with available measurements for absolute LDL-C change were *n* = 32/29, *n* = 33/26, *n* = 27/21, *n* = 15/18, *n* = 11/12, and *n* = 8/8 for patients with/without statin therapy at 3, 9, 15, 21, 27, and 33 months, respectively. Corresponding numbers for percentage LDL-C change were *n* = 31/28, *n* = 32/26, *n* = 26/21, *n* = 15/18, *n* = 11/12, and *n* = 8/8

**Fig. 2:**
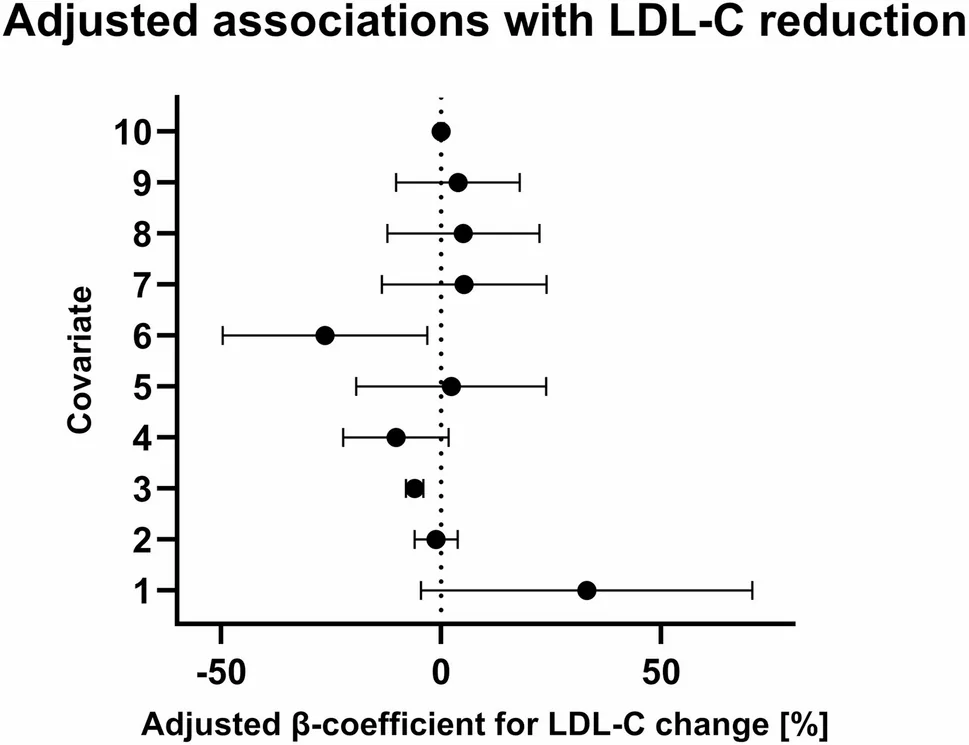
Adjusted associations with LDL-C reduction. Forest plot showing adjusted β coefficients with 95% confidence intervals from the multivariable longitudinal linear mixed-effects model. The dependent variable was percentage LDL-C change from baseline, with negative values indicating greater LDL-C reduction. Patient ID was included as a random intercept to account for repeated measurements within individuals. Covariates were coded as follows: 1: no statin vs. statin; 2: statin intensity per 10% maximum approved dose; 3: baseline LDL-C per 10 mg/dL; 4: age per 10 years; 5: female sex vs. male; 6: no familial hypercholesterolemia vs. familial hypercholesterolemia; 7: no ezetimibe vs. ezetimibe; 8: no bempedoic acid vs. bempedoic acid; 9: no change in background lipid-lowering therapy vs. change compared with the previous visit; and 10: follow-up time per month. The vertical reference line indicates β = 0

The LDL-C profile of patients with and without concomitant statin therapy is presented in Table 2, and longitudinal LDL-C changes from baseline are shown in Fig. 1. Absolute LDL-C concentrations were numerically lower among patients receiving statin therapy across follow-up and reached statistical significance at 21 months (80.0 [60.5–92.0] vs. 103.0 [79.0–135.0] mg/dL; *p* = 0.039) and 27 months (70.0 [63.0–81.0] vs. 97.0 [75.5–120.0] mg/dL; *p* = 0.042). However, when LDL-C response was assessed as absolute or percentage change from baseline, no statistically significant between-group differences were observed at any follow-up time point. As shown in Tables 1 and 45.9% of patients receiving statin therapy were treated with atorvastatin and 45.9% with rosuvastatin, while the remaining 8.2% received less potent statins. Statin intensity was also analyzed. Defining 100% as the maximum approved dose according to the prescribing information, the mean statin intensity was 60.1 ± 33.3%. Regarding statin stability, 13 patients (35.1%) had a change in statin therapy; 11 switched to a different statin, and 2 underwent dose reduction. Adjusted associations are shown in Fig. 2.

**Table 2:** Longitudinal LDL-C profile stratified by concomitant statin therapy

Time point	LDL-C with statin therapy [mg/dL]	LDL-C without statin therapy [mg/dL]	*p*	LDL-C change with statin therapy [%]	LDL-C change without statin therapy [%]	*p*
Baseline	107.5 (93.2–133.2)	128.5 (95.2–183.2)	0.160	—	—	—
3 months	80.5 (53.8–122.2)	96.0 (76.0–122.0)	0.189	−30.9 (− 48.8 to 2.7)	−28.7 (− 43.7 to − 8.3)	0.933
9 months	79.0 (62.0–127.0)	115.5 (81.8–137.5)	0.066	−26.1 (− 48.5 to 11.6)	−25.1 (− 41.3 to − 7.9)	0.749
15 months	74.0 (61.0–98.0)	106.0 (72.0–124.0)	0.284	−24.9 (− 48.0 to 7.4)	−32.8 (− 53.7 to − 12.8)	0.708
21 months	80.0 (60.5–92.0)	103.0 (79.0–135.0)	0.039	−34.1 (− 46.8 to 1.5)	−32.3 (− 46.7 to 38.0)	0.651
27 months	70.0 (63.0–81.0)	97.0 (75.5–120.0)	0.042	−34.8 (− 41.3 to − 28.0)	−30.5 (− 47.3 to 47.8)	0.460
33 months	65.0 (43.0–96.0)	94.5 (66.2–126.8)	0.103	−39.5 (− 56.4 to − 30.9)	−39.1 (− 62.8 to 75.4)	0.645

Values are presented as median with interquartile range. Between-group comparisons were performed using the Mann–Whitney U test. LDL-C change refers to percentage change from baseline. Negative values indicate LDL-C reduction. *p* values are reported to three decimal places. The number of available observations varied across follow-up visits and is reported in Fig. 1

A multivariable longitudinal linear mixed-effects model was fitted to evaluate factors associated with percentage LDL-C reduction over time. Concomitant statin use was not significantly associated with percentage LDL-C reduction (β for no statin vs. statin: 33.1% points; 95% CI − 4.6 to 70.7; *p* = 0.085). Statin intensity was also not significantly associated with LDL-C reduction (β − 0.11 per percentage point of maximum approved dose; 95% CI − 0.60 to 0.37; *p* = 0.644). Baseline LDL-C was strongly associated with percentage LDL-C reduction (β − 0.60 per mg/dL; 95% CI − 0.80 to − 0.41; *p* < 0.001). Familial hypercholesterolemia was associated with a smaller percentage LDL-C reduction (β for no familial hypercholesterolemia vs. familial hypercholesterolemia: −26.4% points; 95% CI − 49.6 to − 3.1; *p* = 0.027), whereas concomitant ezetimibe, bempedoic acid, and changes in background lipid-lowering therapy were not significantly associated with LDL-C reduction.

At 9 months, 10 of 59 patients (16.9%) achieved a ≥ 50% reduction in LDL-C. In the unadjusted responder analysis, responder rates were numerically higher among patients receiving concomitant statin therapy than among those without statin therapy (6/23 [26.1%] vs. 4/36 [11.1%]); however, this difference was not statistically significant (Fisher’s exact test, *p* = 0.166).

For the 9-month responder endpoint, we performed a reduced multivariable logistic regression model adjusted for baseline LDL-C and familial hypercholesterolemia. In this model, concomitant statin therapy was associated with higher odds of achieving ≥ 50% LDL-C reduction (OR 7.69, 95% CI 1.28–45.45; *p* = 0.026). Baseline LDL-C was not significantly associated with responder status (OR 1.01 per mg/dL, 95% CI 1.00–1.03; *p* = 0.126). An extended exploratory logistic regression model for the same 9-month responder endpoint additionally included age, sex, ezetimibe use, and bempedoic acid use; this model yielded imprecise estimates with wide confidence intervals and was therefore interpreted cautiously.

To assess the potential impact of attrition bias, we performed a sensitivity analysis excluding patients who discontinued inclisiran during follow-up. In this analysis, concomitant statin use was significantly associated with percentage LDL-C reduction (β for no statin vs. statin: 72.3% points; 95% CI 24.9 to 119.7; *p* = 0.003), suggesting a stronger LDL-C reduction among statin-treated patients who remained on inclisiran. Statin intensity remained not significantly associated with LDL-C reduction (β 0.35 per percentage point of maximum approved dose; 95% CI − 0.25 to 0.94; *p* = 0.243).

## Discussion

In this real-world cohort, inclisiran therapy was associated with sustained LDL-C reductions in patients with and without concomitant statin background. Descriptive longitudinal analyses showed broadly comparable absolute and percentage LDL-C reductions from baseline between groups. Importantly, the primary adjusted mixed-effects model did not demonstrate a statistically significant association between concomitant statin use or statin intensity and continuous percentage LDL-C reduction. Exploratory responder and attrition sensitivity analyses suggested a possible association between statin background and a more pronounced LDL-C response. However, these secondary findings should be interpreted with caution, particularly given the small sample size, the limited number of responders, wide confidence intervals, and the sensitivity of the estimates to treatment discontinuation. Thus, while inclisiran-induced LDL-C lowering was observed irrespective of statin background, a clinically relevant benefit of concomitant statin therapy cannot be excluded.

Overall LDL-C reduction in our cohort ranged between − 25% and − 40% across follow-up visits, which was lower than the approximately 50% placebo-adjusted reductions reported in the ORION phase III trials. Contemporary real-world evidence provides important context for these findings. The large prospective multicenter CHOLINET Registry evaluated inclisiran in routine clinical practice in Italy and reported robust LDL-C lowering and high LDL-C target achievement, particularly among very-high-risk patients receiving background statin and ezetimibe therapy [7]. CHOLINET observed greater LDL-C reductions and higher target attainment in patients receiving inclisiran on a background of statin therapy, predominantly combined with ezetimibe. However, the specific contributions of statin use and statin intensity were not assessed separately. In contrast, in our cohort, neither concomitant statin use nor statin intensity was significantly associated with the magnitude of LDL-C reduction during inclisiran therapy. This apparent discrepancy may be explained by differences in treatment classification and analytical approach, as CHOLINET compared combined background lipid-lowering regimens rather than isolating the effects of statin exposure and statin intensity. Since statins were predominantly administered in combination with ezetimibe in CHOLINET, the greater LDL-C reduction observed in this group cannot be attributed to statin therapy alone. Our findings therefore provide a complementary statin-stratified analysis to the broader CHOLINET observations by suggesting that, when statin use and intensity are considered separately, neither appears to be a major determinant of the LDL-C response to inclisiran in routine clinical practice.

The clinical relevance of optimizing lipid-lowering strategies is further supported by the BRING-UP Prevention Study, a large prospective multicenter real-world initiative including 4,790 patients with prior atherothrombotic events. In this study, LDL-C target achievement < 55 mg/dL increased from 33.0% to 58.1% after 6 months following structured educational and treatment-optimization efforts, while statin use reached 94.9% and ezetimibe use 84.0% at follow-up. Nevertheless, a substantial proportion of patients remained above guideline-recommended LDL-C targets [8]. Similarly, a Monte-Carlo simulation within the ITACARE-P registry suggested that structured, guideline-based intensification of lipid-lowering therapy, including combination treatment and inclisiran, could substantially improve LDL-C goal attainment in secondary prevention [9]. Together, these studies demonstrate that structured optimization and intensification of lipid-lowering therapy can substantially improve LDL-C target achievement in routine clinical practice, supporting the clinical importance of maintaining and escalating background lipid-lowering therapy whenever feasible.

From a mechanistic perspective, statin-induced upregulation of hepatic PCSK9 expression via SREBP-2 activation provides a biological rationale for a potentially enhanced LDL-C response to PCSK9-targeted therapy in statin-treated patients. However, in our analyses, concomitant statin use and statin intensity were not significantly associated with continuous percentage LDL-C reduction [10–12]. The responder and attrition sensitivity analyses are compatible with a possible role of statin background in selected patients, but these findings are exploratory and should be considered hypothesis-generating rather than confirmatory.

Notably, 27% of patients discontinued inclisiran, predominantly due to insufficient LDL-C reduction. More than half of those switched to PCSK9 monoclonal antibodies achieved LDL-C targets within three months, underscoring interindividual variability and the need for early reassessment and therapeutic escalation in very-high-risk patients.

The present study has several limitations. First, it was retrospective, single-center, and based on a selected outpatient lipidology population, which limits generalizability. Second, the sample size was relatively small and the number of available observations progressively decreased during longer follow-up. Although the longitudinal mixed-effects model allowed adjustment for repeated measurements and clinically relevant confounders, the number of fixed effects was relatively large in relation to the cohort size and follow-up attrition. Therefore, model overfitting and unstable parameter estimates cannot be excluded, and the adjusted estimates should be interpreted as exploratory rather than confirmatory. Third, the responder analysis was based on only 10 patients achieving the ≥ 50% LDL-C reduction endpoint at 9 months. This low number of events resulted in wide confidence intervals and limits the reliability of the estimated odds ratio, despite statistical significance in the reduced logistic regression model. Fourth, discontinuation of inclisiran in a substantial proportion of patients may have introduced attrition bias, particularly because discontinuation was frequently related to insufficient LDL-C reduction. The marked change in the estimated association between statin therapy and LDL-C reduction after excluding discontinuers further indicates that the findings are sensitive to the handling of attrition. Fifth, although blood sampling was performed in the morning under fasting conditions as part of standardized outpatient lipid clinic care, minor variations inherent to the retrospective routine-care setting cannot be excluded. Therefore, the responder and sensitivity analyses should be considered exploratory.

In clinical terms, our data suggest that inclisiran-induced LDL-C lowering can be achieved in patients both with and without concomitant statin therapy. The descriptive LDL-C reductions were broadly comparable between groups, supporting the use of inclisiran also in patients who are not receiving statins. However, the present study does not provide confirmatory evidence that concomitant statin therapy independently modifies the magnitude of inclisiran-associated LDL-C reduction. Exploratory responder and attrition sensitivity analyses raise the hypothesis that statin background may contribute to a more pronounced LDL-C response in selected patients, but this requires confirmation in larger randomized controlled trials.

## Data Availability

Data available upon reasonable request.

## Abbreviations

ASCVD: Atherosclerotic cardiovascular disease
BMI: Body mass index
CI: Confidence interval
HDL: C–High–density lipoprotein cholesterol
IQR: Interquartile range
LDL: C–Low–density lipoprotein cholesterol
Lp(a): Lipoprotein(a)
PCSK9: Proprotein convertase subtilisin/kexin type 9
siRNA: Small interfering Ribonucleic acid
SREBP: 2–Sterol regulatory element–binding protein 2
